# Achieving High Efficiency and High Throughput in Erasure Code-Based Distributed Storage for Blockchain †

**DOI:** 10.3390/s25072161

**Published:** 2025-03-28

**Authors:** So-Hyun Park, So-Yeon Kim, So-Hui Kim, Il-Gu Lee

**Affiliations:** Department of Future Convergence Technology Engineering, Sungshin Women’s University, Seoul 02844, Republic of Korea; 220227022@sungshin.ac.kr (S.-H.P.); 220237014@sungshin.ac.kr (S.-Y.K.); 220226034@sungshin.ac.kr (S.-H.K.)

**Keywords:** automatic repeat request, blockchain, data recovery, distributed storage, erasure code, wireless sensor networks, trigger

## Abstract

A blockchain is a decentralized peer-to-peer network in which all nodes store data in copies, ensuring data integrity, as transactions cannot be changed or deleted. This can lead to duplicate data storage, resulting in high storage overhead, especially in storage-constrained environments, such as the Internet of Things (IoT) or sensor systems. Distributed storage techniques utilizing erasure code (EC) have been investigated to address this issue. Although EC-based blockchain storage increases storage efficiency, encoded chunks distributed across multiple nodes must be received to restore and access the original blocks. However, studies on increasing the data transmission efficiency of EC-based blockchain storage are limited. In this study, we propose a data transmission technique called trigger-based automatic repeat request (ARQ), enabling stable data recovery while ensuring low latency and high-throughput performance, even with frequent node failure. This technique increased the throughput efficiency by 8% while maintaining the decentralization of the blockchain. Furthermore, it maximized the storage efficiency of EC-based distributed blockchain storage by >99.8%, while solving the recovery overhead problem due to data transmission. Using the trigger-based ARQ scheme with an EC-based distribution technique, blockchains can reduce storage overhead while effectively accessing the original blocks, overcoming the limitations of conventional EC-based distributed storage.

## 1. Introduction

Web 3.0, which has gained increasing attention as the next generation of the World Wide Web, is an intelligent decentralized web system that can generate and obtain data from individuals. This generation provides a semantic web environment that provides customized information for individual situations and contexts [1]. Although the concept of Web 3.0 is still evolving and implementation technology has not yet matured, decentralized storage, which is enabled by several technologies, such as blockchain technology, is essential to implement the main objective of Web 3.0 [2]. The metaverse, which is a representative service that utilizes Web 3.0, requires considerable computing and storage resources for storing content [2]. A blockchain, which is a type of ledger, has been utilized in various storage-based technologies to ensure data integrity and reliability. Owing to the growth of hyperconnected and superconvergence systems such as the Internet of Things (IoT), the amount of data stored in different systems such as blockchains has increased [3,4].

Moreover, blockchain is utilized in networks such as the IoT and sensors to ensure data security [5,6]. Sensors that collect and store data often handle private and sensitive information, making it crucial to guarantee data confidentiality and integrity. Blockchain operates based on hashing and ensures data integrity while enabling data traceability, making it a key technology for securing data in wireless sensor networks [7,8].

Because blockchains must ensure reliability and transparency, each participating node stores a copy of the entire blockchain, resulting in an exponentially increasing storage burden [3]. With the participation of more nodes, the storage overhead increases linearly, and more blocks are generated in traditional full-node-based blockchain storage systems that replicate all the blocks per node [3]. The requirements associated with large amounts of storage can significantly hamper the operation of blockchains on resource-constrained systems, such as the IoT [9].

Lightweight blockchain storage techniques, such as the light node technique [10], which stores only the block header information of the blockchain, or transaction pruning [11], which deletes old transactions and stores only the latest transactions, have been considered. Each technique requires a separate full node that stores all the blocks, which can compromise the decentralization of a blockchain network. While ensuring the decentralization of blockchain networks, methods that reduce blockchain storage requirements must ensure the decentralization of blockchain networks while reducing the storage burden of the participating nodes. Distributing blockchain transaction data using coding has emerged as a representative method for reducing storage overheads.

Erasure code (EC)—a technique used for the distributed storage of blockchain transaction data—can recover the original data even if part of the data is lost. It divides the original data into k pieces and adds m parity symbols (k>m) to generate n encoded chunks [2]. When using the Reed Solomon (RS) code, up to m errors can be detected and up to m/2 errors can be corrected among n encoded chunks [2].

Because n nodes store n encoded chunks individually, the EC-based distributed storage method can store data that are resistant to m node failures while reducing the storage overhead by 1/k compared with conventional full-node-based storage. EC-based storage technology is being studied as a lightweight blockchain storage method that addresses the dependency and reliability issues of the traditional InterPlanetary File System and cloud-based systems. Although many studies have investigated various methods for applying an EC to blockchain storage, most have rarely considered block recovery or the cost of recovering entire distributed encoded chunks of the original blocks [12,13,14,15,16]. In [13], multiple encoded chunk copies were redundantly stored to ensure the recovery of the original data and reduce the recovery cost. However, the storage overhead increased because of the redundant encoded chunks. Additionally, optimization algorithms proposed to reduce storage data redundancy have not considered the recovery performance [12,13,14,15,16]. To use EC-based distributed storage more efficiently, block recovery performance is a critical issue; thus, the recovery mechanism should be treated as an important consideration [17].

This study proposed an effective recovery method called the trigger-based automatic repeat request (ARQ) technique for EC-based distributed blockchain storage. This technique can reduce the communication cost of recovering encoded original blocks, even in environments with node failures.

The main contributions of this study are as follows:

- The trigger-based ARQ method, which uses trigger signals much smaller than the encoded chunks, reduces the data transmission overhead for recovering the original blocks from EC-based distributed blockchain storage while maintaining the decentralization of the blockchains.
- The proposed trigger-based ARQ technique enables stable data recovery while ensuring low latency and high-throughput performance, even when node failure is frequent.
- Using the trigger-based ARQ scheme with an EC-based distribution technique, blockchains can reduce storage overhead while effectively accessing the original blocks, overcoming the limitations of conventional EC-based distributed storage.

The remainder of this paper is organized as follows. Section 2 examines previous studies on reducing the data-recovery costs of EC-based distributed blockchain storage. Section 3 explains the system model of EC-based distributed blockchain storage and existing efficient block recovery methods. Section 4 presents the evaluation of the performance of non-trigger and trigger-based ARQ methods in terms of total data transmission time and throughput. Finally, Section 5 concludes this paper.

## 2. Related Work

Blockchain systems employ a replication-based storage method in which every node stores the same data to ensure storage reliability. EC is an alternative approach to reduce the storage burden on nodes, which must store large amounts of data [18]. This code can achieve high stability and low overhead, leading to high data-recovery costs. Research has been conducted to improve the recovery efficiency, mainly by suggesting adjustments to the chunk size or physical distance [19,20] or proposing new coding methods to improve the data recovery performance [21,22,23]. Most studies have focused only on data storage efficiency and performance [13,24,25], whereas only a few have considered data recovery from a network perspective.

Qiu et al. [21] proposed a hybrid EC-fusion method that integrates RS code with minimum storage regeneration (MSR) code, aiming to optimize storage and recovery costs. By leveraging MSR coding when frequent decoding is required, this method demonstrated improved application response time and recovery performance compared to traditional MSR, RS, and Local Reconstruction Codes. However, a key limitation of this approach is its narrow evaluation scope—its performance was assessed only for the failure recovery of a single chunk. In real-world blockchain environments where multiple chunk failures are common owing to distributed storage characteristics and network fluctuations, the effectiveness of this hybrid fusion method remains uncertain. Furthermore, this method does not explicitly address the network overhead introduced during the recovery process, which can significantly affect the overall system performance.

Liu et al. [22] proposed a Z code that optimizes both recovery bandwidth and storage efficiency. By eliminating the exclusive OR (XOR) code attribute and modifying it into a generalized Z code, the scheme achieves the maximum separable distance (MSD) attribute. Although this approach enhances data-recovery efficiency under normal circumstances, it has notable limitations. Specifically, its performance degrades significantly in scenarios involving multiple node failures, which are common in large-scale distributed blockchain storage systems. Additionally, the study did not thoroughly address network-related overhead, which plays a crucial role in recovery performance, particularly when data chunks must be retrieved from distributed nodes.

Caneleo et al. [23] proposed an XOR-based coding scheme designed to improve the recovery efficiency of storage systems spread across multiple regions. Their method requires fewer parity blocks for data recovery than RS-like codes, making it an attractive solution for reducing bandwidth consumption. However, this improvement comes at the cost of increased storage overhead, as it generates more parity blocks than the standard RS code. This trade-off raises concerns regarding long-term scalability in blockchain environments, where storage efficiency is critical. Moreover, although the study demonstrated enhanced recovery performance, it did not fully consider network congestion and latency, which can significantly affect real-world recovery times in blockchain-based distributed storage systems.

Shan et al. [19] improved the data recovery performance by adjusting the chunk sizes. Furthermore, they improved the recovery performance of code regeneration using variable chunk sizes by 1.85 times that of the RS code scheme while maintaining a low level of read-time degradation. Regenerating codes are ECs that minimize the data required for recovery and are recovered in chunk units rather than bytes. However, although their approach improves the recovery speed, it does not fully account for the impact of network congestion, which can significantly affect the recovery performance in real-world distributed blockchain environments. Additionally, adjusting chunk sizes requires fine-tuning based on workload characteristics, making it less adaptable to dynamic blockchain storage conditions.

Song et al. [20] enhanced the recovery efficiency using a low-cost node selection strategy that selects nodes with physically short distances and low loads. Additionally, they stored the RS parity symbols in the data node, thereby reducing cross-rack traffic during recovery. The data-recovery time was less than 42.41% compared to that under the RS code scheme and less than 36.58% compared to that under deterministic data distribution. Despite a reduction in the transmission and recovery time between the server racks, the amount of data transmission and storage costs within some racks still increased.

Meng et al. [24] and Qi et al. [13] proposed EC-based blockchain storage techniques to reduce the storage burden on blockchain nodes. Qi et al. [13] introduced BFT-Store, a novel storage engine that integrates EC and Byzantine Fault Tolerance (BFT) to enhance storage scalability in permissioned blockchains. Although this approach effectively saves storage space and strengthens reliability, it also has the drawback of increasing network overhead. Meng et al. [24] proposed an EC-based blockchain storage method, focusing on reducing storage overhead. However, their approach suffers from the limitation of high computational overhead for data recovery. EC-based recovery typically involves complex decoding operations, which can be computationally expensive, particularly for resource-constrained blockchain nodes. Although their work provides storage efficiency benefits, it does not sufficiently address the trade-off between computational cost and recovery speed, making it less suitable for high-frequency data retrieval scenarios.

Zhang et al. [25] addressed data loss issues caused by malicious nodes in permissioned blockchains by proposing a dynamic data recovery scheme based on Local Reconstruction Codes (LRC). However, because LRC coding and recovery rely on node reputation, cooperation among nodes is required, which is a drawback. Additionally, this approach does not fully address lightweight storage and complete data recovery.

Although these studies contributed to EC-based blockchain storage, they primarily focused on storage efficiency and fault tolerance, with limited consideration of network communication overhead and recovery performance in large-scale blockchain environments. Future research should address these challenges by optimizing recovery mechanisms while minimizing network traffic and computational costs.

## 3. System Model

### 3.1. Erasure Code-Based Distributed Blockchain Storage

RS code can detect up to m errors by adding m parity symbols to k original data and correcting up to m/2 errors. When k blocks are stacked, m parities are added to encode the (n, k) RS code and generate n (n>k) encoded chunks. The storage overhead of each node is reduced to 1/k, because every n node stores only one out of n blocks one by one, whereas the full-node-based blockchain nodes store all n blocks.

**Figure 1:**
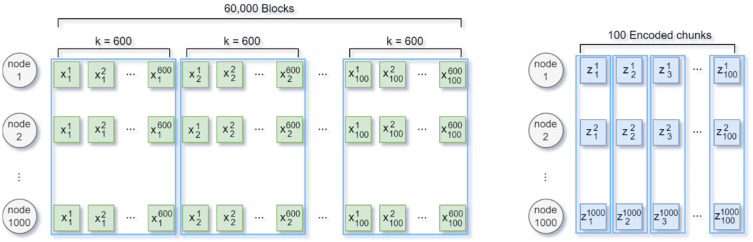
Full-node-based blockchain storage and EC-based distributed blockchain storage.

In an encoding round in which k blocks are stacked and encoded by applying an (n, k) RS code, $x_{r}^{i}\;(1\leq i\leq k)$ refers to the ith block of the rth encoding round, and $z_{r}^{j}(1\leq j\leq n)$ refers to the jth encoded chunk of the rth encoding round. As shown in Figure 1, 60,000 blocks were distributed and stored by applying the (1000, 600) RS code 100 times to a blockchain network of 1000 nodes. All 1000 nodes in the existing full-node-based blockchain stored 60,000 pieces of data equally. When 600 blocks from $x_{1}^{1}$ to $x_{1}^{600}$ were stacked, 400 parity symbols were added to apply the (1000, 600) RS code to generate 1000 encoded chunks from $z_{1}^{1}$ to $z_{1}^{1000}$. The encoded chunks $z_{1}^{1}$ to $z_{1}^{1000}$ were distributed and stored individually in 1000 nodes. After repeating RS encoding 100 times, the 60,000 blocks were reduced to 100 encoded chunks for a single node, reducing the storage space burden to 1/600.

### 3.2. Effective Block Recovery

At least k encoded chunks must be received to recover original EC-based distributed blocks. Because each node stores a single encoded chunk, the fastest method is to recover the original block by receiving k−1 chunks from the other nodes. However, if a node cannot correctly deliver the data, the encoded chunks are retransmitted until the destination node receives the data correctly. If these data are not transmitted correctly, ARQ is used to detect errors and request retransmissions. However, as more errors occur under this method, the data transmission time required to recover the original block increases, and the throughput and energy efficiency are degraded.

Encoded chunks require a long transmission time because of their large data size. In this study, instead of the conventional ARQ technique that retransmits long data packets, we propose a trigger-based ARQ scheme that exchanges only trigger signals. An original block can be recovered faster and more effectively using a trigger signal smaller than the encoded chunk. The link status can be recognized first through the trigger signal, and the encoded chunk can be transmitted with high throughput by ensuring the k−1 normal node necessary for recovery.

**Figure 2:**
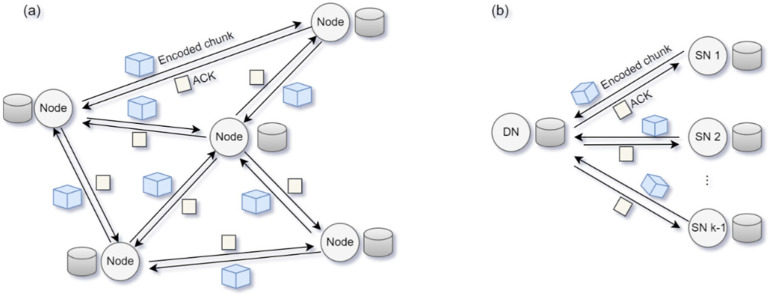
(**a**) Data transmission in a decentralized peer-to-peer (P2P) blockchain network and (**b**) encoded chunk transmission between multiple source nodes (SNs) and a single destination node (DN).

All nodes must receive encoded chunks from other nodes to recover the original blocks; however, there is no protocol for controlling the order or flow of transmission in a decentralized network. All the distributed nodes in a blockchain are source nodes (SNs) and destination nodes (DNs) that simultaneously transmit and receive data, respectively. In real-world distributed networks, multiple nodes simultaneously receive and request data, as shown in Figure 2a, resulting in congestion and many potential collisions. Many other considerations are available for transmitting data at a low latency while occupying limited channel resources fairly without conflicts. For example, in a network in which data transmission occurs through distributed competition, the time duration for channel access should be allocated to all the nodes [26]. In this study, we propose a method for receiving data with a low delay when one node obtains the time duration for data reception, as shown in Figure 2b. Through channel occupancy competition, a DN receives an encoded chunk from the remaining k−1 SNs. If all the k−1 SNs send encoded chunks without failure, the original block can be recovered without retransmission. However, if the encoded chunk sent by SN2 is incorrectly transmitted, and the DN does not send an ACK, SN2 needs to send that chunk again, resulting in the original block recovery process being delayed.

Various errors can occur during blockchain data transmission. For example, if the data stored on a node are lost, a malicious Byzantine node can send incorrect data or the received signals can be degraded through network interference, collision, or noise during transmission. In this study, all data transmission failures, such as nodes abnormally operating or leaving the blockchain network, were assumed to be errors caused by node failure. Additionally, even if a failed node did not correctly transmit data, it could presumably become a normal node through several retransmission attempts and correctly transmit the data.

**Figure 3:**
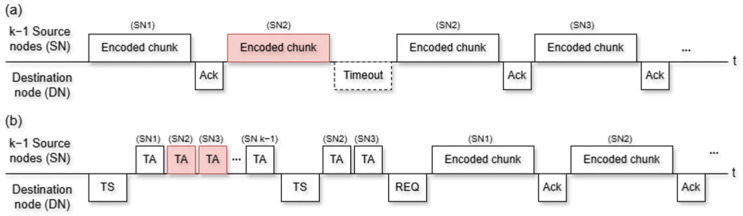
Data transmission process of (**a**) non-trigger ARQ and (**b**) trigger-based ARQ.

Figure 3a,b compare the operating methods of the non-trigger ARQ and trigger-based ARQ techniques, respectively. The key difference between the conventional method and the proposed method is that the trigger-based ARQ scheme exchanges a trigger signal in advance instead of directly transmitting the actual data. This allows the system to retransmit only the trigger signal rather than the actual data, effectively reducing the overhead. The non-trigger ARQ receives encoded chunks sequentially from k−1 SNs. If SN2 does not correctly transmit the data, and an ACK is not received from the DN, as shown in Figure 3a, SN2 sends the encoded chunk again and waits for the ACK. If SN2 receives an ACK, SN3 sends the encoded chunk. All nodes can continuously detect whether a channel is in use or transmit data sequentially by computing the link reservation time. More encoded chunks are retransmitted as the number of node failures increases, resulting in an increase in the total data transmission time and deterioration in the throughput, which represents the actual number of encoded chunks transmitted within a unit of time.

The trigger-based ARQ technique shown in Figure 3b can reduce the overhead caused by data retransmission, which is a disadvantage of the non-trigger ARQ technique, by retransmitting only the trigger acknowledgment (TA), using the trigger signal (TS) proposed in this study. The DN selects k−1 SNs to receive data and includes these SNs in the TS group address field for broadcasting. The SNs that receive the TS send the TA sequentially. If the TA does not arrive at the DN correctly, the DN sends the TS back to the SN to receive the TA. If more than k−1 normal TAs are received, the DN sends a REQ to the k−1 among the normal SNs to request data and, finally, starts to receive the encoded chunks sequentially. As the normal link operation of each node was confirmed through the trigger-based ARQ, the recovery time could be shortened by reducing the number of encoded chunk retransmissions due to node failure.

The trigger-based ARQ technique reduced the data transmission time needed to recover EC-based distributed blockchain storage. In an environment with node failures, this technique can improve the overall throughput of data transmission compared to the non-trigger ARQ technique, which performs extensive data retransmission. If the link conditions at the time of triggering remain the same, even an encoded chunk can be quickly transmitted with a low error probability. The data for link congestion control are small because they do not contain much information and are transmitted at low data rates for accurate transmissions, even in situations with potentially high network errors. However, because the data containing actual information are large, they are transmitted at fast data rates to improve throughput, thereby impairing the signal-to-noise ratio of the reception. Therefore, when the trigger-based ARQ is applied to transmit a long-encoded chunk, network interference, reception signal degradation owing to loss, and data loss may occur because of noise.

Nevertheless, to recover the original data block of the distributed blockchain, guaranteeing at least k normal nodes that can stably transmit encoded chunks is essential. The trigger-based ARQ technique, which identifies normal nodes faster than the non-trigger ARQ technique does, shortens the data recovery process.

## 4. Evaluation and Analysis

We compared the throughput of data transmission under the non-trigger and trigger-based ARQ methods when distributed encoded chunks were being transmitted from each SN to the DN to recover the original blockchain data. Moreover, the recovery cost and processing performance under the proposed trigger-based ARQ data transmission method were compared with those under different blockchain storage methods: conventional full-node-based storage, redundant storage based on the repetition code (RC), and EC-based distributed storage. The trigger-based ARQ technique could address the limitations of EC-based distributed storage by simultaneously reducing storage and recovery overheads.

### 4.1. Experimental Environment

A performance evaluation was conducted through numerical analysis based on simulations. The simulation were implemented using Python version 3.10.8. Sixty thousand original blocks were RS-encoded and stored in a blockchain network of one thousand nodes to evaluate the performance of the trigger-based ARQ, as shown in Figure 1. The data transmission time and throughput when one DN received data from k−1 SNs to recover the original blocks were compared. Table 1 lists the number of total nodes and normal nodes, the number of encoded chunks per node, the encoded chunk size, the ACK size, the trigger signal size, and the data rate in the experimental environment. The size of the encoded chunk was set to 1 KB, and those of the ACK and trigger signals were set to 14 and 20 bytes, respectively. The trigger signal and encoded chunk were assumed to be transmitted at the same data transmission rate of 5 Mbps.

This study evaluates the performance of a Practical Byzantine Fault-tolerant network, where up to one-third of the total nodes (B=⌊(n−1)/3⌋) may experience failures. To model the node failure rate, we assume that the number of faulty nodes follows a Poisson distribution with a mean failure rate of λ. The expected number of Byzantine nodes, denoted as E[X], is given by the following:

(1) $$E\left[X\right]=\lambda \;.$$

The maximum node failure rate (${FR}_{max}$) can be computed as follows:

(2) $${FR}_{max}=\frac{\lambda }{n}\times 100$$

where λ varies from 0 to 200 in increments of 20, ensuring that the node failure rate remains within 0–20% of the total nodes.

In the absence of node failures, data are received sequentially from *k* = *N* − *B* normal nodes without retransmissions, achieving maximum throughput. However, as the node failure rate increases, additional retransmissions are required, leading to an increased transmission time and reduced throughput. Moreover, node failures are not static but change dynamically over time as data transmission progresses. In each network variation cycle, the number of faulty nodes is updated, reflecting real-world scenarios in which network conditions fluctuate owing to congestion, interference, or hardware malfunctions. This variation in the failure rate affects the number of retransmissions required, leading to fluctuations in transmission time and throughput. In the simulation, the node failure rate (FR) was updated approximately every 10 data transmissions, ensuring that the network conditions evolve over time. The failure rate (*FR*) fluctuates between a minimum of 0% and a maximum of ${FR}_{max}$, depending on the Poisson-distributed failure model.

Under the non-trigger ARQ technique, the SN transmits an encoded chunk to the DN, which responds via an ACK. If a node failure occurs and an ACK is not received, the SN retransmits an encoded chunk, attempting it up to eight times. If data transmission fails even after this, a new request for data is sent to another SN to initiate the ARQ-based data transmission. The trigger-based ARQ technique is a transmission technique in which the DN sends a trigger signal to the SN, receives a trigger ACK from the SN to check the link status in advance, and then receives encoded chunks from k−1 SNs. The trigger ACK is retransmitted up to eight times, and the DN sends a trigger signal to another SN if the retransmission fails to ensure the reliability of the k−1 SNs and then receives an encoded chunk.

### 4.2. Throughput Evaluation

The throughputs when transmitting an entire encoded chunk were compared based on the node failure rate. The throughput was the total transmission time compared to the number of bits of the encoded chunks that needed to be transmitted (Equation (3)).

(3) $$Throughput\left(Mbps\right)=\frac{dataNum\times dataSize\times k}{Total\;transmission\;time}$$

**Figure 4:**
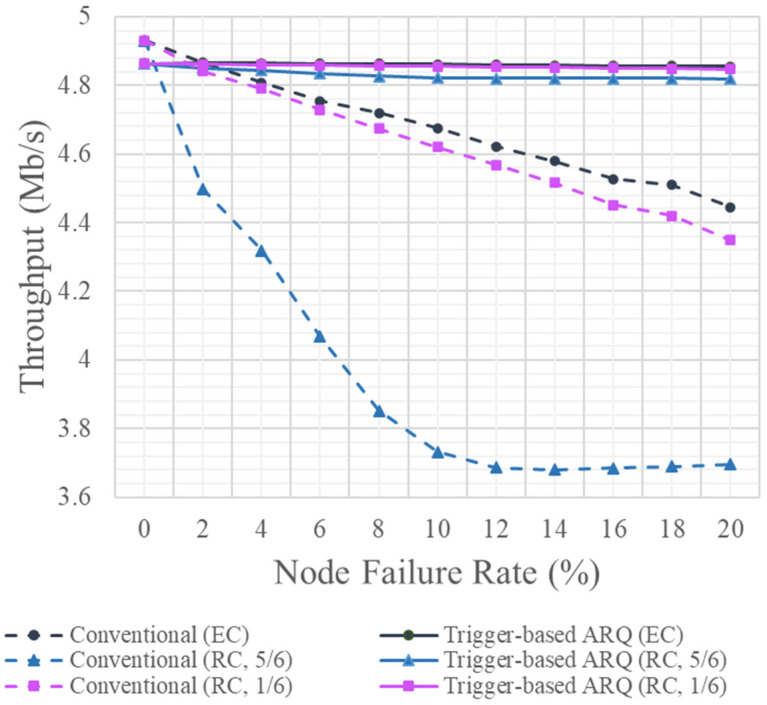
Throughputs under various node failure rates.

Data are not retransmitted in the absence of node failures, resulting in time only being taken to receive data sequentially from k−1 nodes. However, the data transmission time increases as the number of node failures increases, owing to data retransmission. Figure 4 shows the average throughput according to the node failure rate. The node failure rate was 0–20% of the total 1000 nodes, which refers to the case in which the node failed to transmit data. The transmission time for the encoded chunk to recover a total of 60,000 original blocks when using the non-trigger and trigger-based ARQ techniques as the node failure rate increased was simulated, and the throughput deteriorated as a result.

When the node failure rate was 0%, TS and TA acted as overheads. The transmission time under the conventional non-trigger ARQ transmission method, which transmitted only encoded chunks and ACKs, was less than that under the trigger-based ARQ method. The same was observed at a node failure rate of 2%. However, with an increase in the node failure rate, this time increased by more than 10% compared to when there were no node failures, and the delay also proportionally increased.

However, under the trigger-based ARQ technique, the transmission delay owing to retransmission increased by only approximately 0.3% when the node failure rate increased by more than 2%, allowing encoded chunks to be transmitted with a low delay, even during frequent node failures. The throughput deteriorated as the retransmission of the encoded chunks TS and TA increased. At 5 Mbps data transmission, as shown in Table 2, the non-trigger ARQ technique guaranteed a throughput of 4.93 Mbps when there was no node failure, which dropped to 4.44 Mbps when the node failure rate reached 20%. In contrast, when using the trigger-based ARQ technique, the throughput was 4.86 Mbps when no node failure occurred, which was slightly lower than that under the non-trigger ARQ technique; however, the data transmission rate was 4.85 Mbps even when the node failure rate was 20%.

The throughput efficiency of the data transmission was measured using the actual throughput and compared to the transmission data rate when 60,000 blocks were recovered under each transmission technique. Under the non-trigger ARQ technique, the throughput efficiency decreased from 98.6 to 89.6% when the node failure rate was 20%, whereas, under the trigger-based ARQ technique, the throughput efficiency changed from 97.2 to 97.3% at the same node failure rate. The throughput efficiency under the trigger-based ARQ scheme was 8% higher than that under the non-trigger ARQ technique. An increased throughput efficiency was observed when one DN received an encoded chunk from k−1 SNs.

When DNs receive encoded chunks in real-world scenarios, many factors, including in-channel interference, collision, and transmission delay due to the contention for channel access, delay the recovery time. Therefore, the non-trigger ARQ technique requires a longer data transmission time for block recovery. The trigger-based ARQ technique can reduce the original data-recovery time by enabling highly reliable and efficient data transmission, even in crowded networks.

### 4.3. Storage Efficiency Analysis

In full-node-based blockchain storage, each node stores a copy, resulting in data redundancy and, consequently, a vast storage load. However, because all the nodes have blocks, recovering the data to access them is not necessary. Table 2 lists the storage efficiencies under various storage schemes and compares the data transmission throughput under each ARQ scheme at a node failure rate of 20%. The storage efficiency per node is the ratio of the actual stored data size to the total data. To increase the storage efficiency, the RC scheme can be applied instead of storing the entire block. Storing only a portion of the data in copies can save storage space compared to traditional full-node-based storage.

The EC-based storage method encodes 600 blocks into 1000 encoded chunks and divides 1000 nodes individually, whereas the RC-based storage method stores only 600 blocks partially based on the code rate. For example, if the code rate is 1/6, only 100 of the 600 blocks are stored in copies, and the remaining 500 are transmitted from the other nodes to recover the original block. The per-node storage reduction is calculated as follows:

(4) $$Storage\;reduction=\left(1-\frac{Actual\;stored\;data}{Original\;data}\right)\times 100.$$

The effective per-node storage requirement in an EC-based system is 1/600 that of the original data, leading to an overall storage reduction of (1−1/600)×100=99.83%. By contrast, the RC-based method with a code rate of 1/6 stores 100 of the 600 blocks per node, resulting in a per-node storage reduction of (1−100/600)×100=83.33%. Although the throughput of the EC-based scheme and the RC-based scheme (with a code rate of 1/6) is similar, the EC-based scheme achieves approximately six times higher storage efficiency.

## 5. Conclusions

EC-based distributed storage is a storage-efficient blockchain storage method that differs from traditional full-node-based storage, in which all blocks are redundantly stored on each node. Instead, it stores only a portion of the blocks on each node, thereby reducing the storage overhead. However, to retrieve data, the distributed blocks must be reconstructed, which requires the transmission of all the necessary blocks from multiple nodes. This approach has a limitation in that it relies on retrieving blocks from all nodes, which can lead to significant performance degradation in the presence of frequent node failures. To address this issue, an efficient recovery mechanism is required to improve the data retrieval throughput. By applying the proposed trigger-based ARQ scheme, the throughput improves by 8% compared to conventional methods, even in environments where the maximum node failure rate reaches 20%. Additionally, per-node storage usage is reduced by 99.8%. This study evaluates the performance of the proposed scheme by measuring the throughput when a single node retrieves all blocks. In future work, we plan to implement a more complex scenario, where multiple nodes simultaneously retrieve blocks from multiple distributed nodes, rather than a single node retrieving blocks from all other nodes, to further analyze system performance. Additionally, we aim to extend our research by developing recovery algorithms that are adaptable to various blockchain frameworks and coding schemes, including forward error correction techniques. Furthermore, we plan to investigate potential security risks, such as malicious nodes manipulating trigger signals or Byzantine nodes interfering with the ARQ mechanism, and explore mitigation strategies to enhance the security and robustness of the proposed method in distributed blockchain storage systems.

## Figures and Tables

**Table 1:** Performance evaluation parameters.

Parameter	Value
# of total nodes (n)	1000
# of Byzantine nodes (B)	⌊(n−1)/3⌋
# of normal nodes (k)	n−B
# of encoded chunks per node (dataNum)	100
Encoded chunk size (dataSize)	1 KB
ACK, trigger ACK (TA) size	14 Bytes
Trigger signal size	20 Bytes
Data rate	5 Mbps

**Table 2:** Storage efficiencies and data transmission throughputs under various storage schemes.

Storage Scheme	Storage Efficiency	Throughput (Mb/s) at a Node Failure Rate of 20%	
		Non-Trigger ARQ	Trigger-Based ARQ
Full-node-based storage	1	-	-
RC-based distributed storage (code rate of 5/6)	1.2	3.69	4.81
RC-based distributed storage (code rate of 1/6)	6	4.34	4.84
(1000, 600) RS-based distributed storage	600	4.44	4.85

## Data Availability

Data are contained within the article.

## Abbreviations

The following abbreviations are used in this manuscript:

DN	Destination node
EC	Erasure code
IoT	Internet of Things
LRC	Local Reconstruction Codes
MSD	Maximum separable distance
MSR	Minimum storage regeneration
RC	Repetition code
RS	Reed Solomon
SN	Source nodes
TA	Trigger ACK
